# Integrating Plasma pTau‐217 and Aβ42/Aβ40 Ratio for Accurate Non‐Invasive Prediction of Brain Amyloid Accumulation

**DOI:** 10.1002/alz70856_103484

**Published:** 2025-12-26

**Authors:** José Antonio Allué, Leticia Sarasa, María Pascual‐Lucas, Noelia Fandos, Judith Romero, Jorge Loscos, Adrián Sánchez, Jose Terencio, María José Gil‐Moreno, Isabel Ortega‐Madueño, Jordi A Matias‐Guiu

**Affiliations:** ^1^ Araclon Biotech, Zaragoza, Zaragoza, Spain; ^2^ Grifols S.A., Barcelona, Barcelona, Spain; ^3^ Hospital Clínico San Carlos, Madrid, Madrid, Spain; ^4^ Hospital Clinico Universitario San Carlos, Madrid, Madrid, Spain

## Abstract

**Background:**

The identification of individuals with amyloid‐beta (Aβ) brain accumulation is crucial for the early diagnosis and management of Alzheimer's disease (AD). Plasma biomarkers have emerged as promising non‐invasive tools for this purpose. We developed a logistic regression model incorporating plasma pTau‐217, Aβ42/Aβ40 ratio, APOE genotype, and patient age to predict amyloid accumulation in the brain, determined through cerebrospinal fluid (CSF) Aβ42/Aβ40 measurements.

**Method:**

This study included 54 patients from the Cognitive Neurology Unit at the Hospital Clínico San Carlos in Madrid, Spain. Plasma pTau‐217 (Lumipulse Assay, Fujirebio) and Aβ42/Aβ40 (ABtest‐MS, Araclon Biotech) were measured, alongside APOE genotyping and age data collection. Logistic regression models were constructed to predict amyloid status, with model performance evaluated using Akaike Information Criterion (AIC), accuracy, and the area under the receiver operating characteristic curve (AUC). The clinical utility of the model was also assessed by analyzing its sensitivity, specificity, and negative and positive predictive values.

**Result:**

The proposed model incorporating plasma pTau‐217, plasma Aβ42/Aβ40 ratio, APOE genotype, and age demonstrated the best predictive performance, with the lowest AIC and an AUC of 0.97. This model achieved an accuracy of 93%. Importantly, this high level of accuracy suggests that an approach using two cutoff points may not be necessary in initial evaluations, although this could be adapted based on specific clinical priorities. The proposed model yielded Positive and Negative Predictive Values of 96% and 89%, respectively, at a prevalence of 52%. Despite the small cohort size, these findings are highly promising and warrant validation in larger populations.

**Conclusion:**

Our study supports the use of a plasma‐based model incorporating plasma pTau‐217 and Aβ42/Aβ40 ratio, APOE genotype and age, as an effective tool for predicting amyloid brain accumulation. This model not only offers robust statistical performance but also holds strong clinical relevance, providing a non‐invasive approach with high predictive accuracy. These results underscore the potential of plasma biomarkers in advancing AD diagnosis and call for further validation in larger cohorts to confirm their applicability in broader clinical settings.

